# Single-cell transcriptomics using spliced leader PCR: Evidence for multiple losses of photosynthesis in polykrikoid dinoflagellates

**DOI:** 10.1186/s12864-015-1636-8

**Published:** 2015-07-17

**Authors:** Gregory S. Gavelis, Richard A. White, Curtis A. Suttle, Patrick J. Keeling, Brian S. Leander

**Affiliations:** Department of Zoology, University of British Columbia, Vancouver, BC V6T1Z4 Canada; Department of Microbiology & Immunology, University of British Columbia, Vancouver, BC V6T1Z4 Canada; Department of Botany, University of British Columbia, Vancouver, BC V6T1Z4 Canada; Department of Earth, Ocean and Atmospheric Sciences, Vancouver, BC V6T1Z4 Canada

**Keywords:** Chloroplast, Dinoflagellates, Endosymbiosis, Mixotrophy, Peridinin, *Polykrikos*, Spliced leader

## Abstract

**Background:**

Most microbial eukaryotes are uncultivated and thus poorly suited to standard genomic techniques. This is the case for *Polykrikos lebouriae*, a dinoflagellate with ultrastructurally aberrant plastids. It has been suggested that these plastids stem from a novel symbiosis with either a diatom or haptophyte, but this hypothesis has been difficult to test as *P. lebouriae* dwells in marine sand rife with potential genetic contaminants.

**Results:**

We applied spliced-leader targeted PCR (SLPCR) to obtain dinoflagellate-specific transcriptomes on single-cell isolates of *P. lebouriae* from marine sediments. *Polykrikos lebouriae* expressed nuclear-encoded photosynthetic genes that were characteristic of the peridinin-plastids of dinoflagellates, rather than those from a diatom of haptophyte. We confirmed these findings at the genomic level using multiple displacement amplification (MDA) to obtain a partial plastome of *P. lebouriae*.

**Conclusion:**

From these data, we infer that *P. lebouriae* has retained the peridinin plastids ancestral for dinoflagellates as a whole, while its closest relatives have lost photosynthesis multiple times independently. We discuss these losses with reference to mixotrophy in polykrikoid dinoflagellates. Our findings demonstrate new levels of variation associated with the peridinin plastids of dinoflagellates and the usefulness of SLPCR approaches on single cell isolates. Unlike other transcriptomic methods, SLPCR has taxonomic specificity, and can in principle be adapted to different splice-leader bearing groups.

**Electronic supplementary material:**

The online version of this article (doi:10.1186/s12864-015-1636-8) contains supplementary material, which is available to authorized users.

## Background

In recent decades, we have become increasingly aware of the complex history of plastids, with red algae and green algae (including land plants) harnessing “primary” plastids from a single ancient cyanobacterium, and red algae subsequently lending “secondary” plastids to most eukaryotic phytoplankton (e.g., haptophytes, cryptophytes, diatoms, dinoflagellates), and kelps [1, 2]. Some dinoflagellates even possess “tertiary” plastids derived from haptophytes, cryptophytes, and diatoms [3, 4], and other organisms sequester plastids temporarily, with uncertain degrees of integration [5, 6]. Plastid acquisitions can be difficult to study genetically, as even small amounts of contamination can provide false impressions of photosynthetic gene transfer to the host’s nuclear genome. This is further complicated by the fact that many of these organisms are rare, unicellular, and have yet to be cultured in lab.

**Fig. 1:**
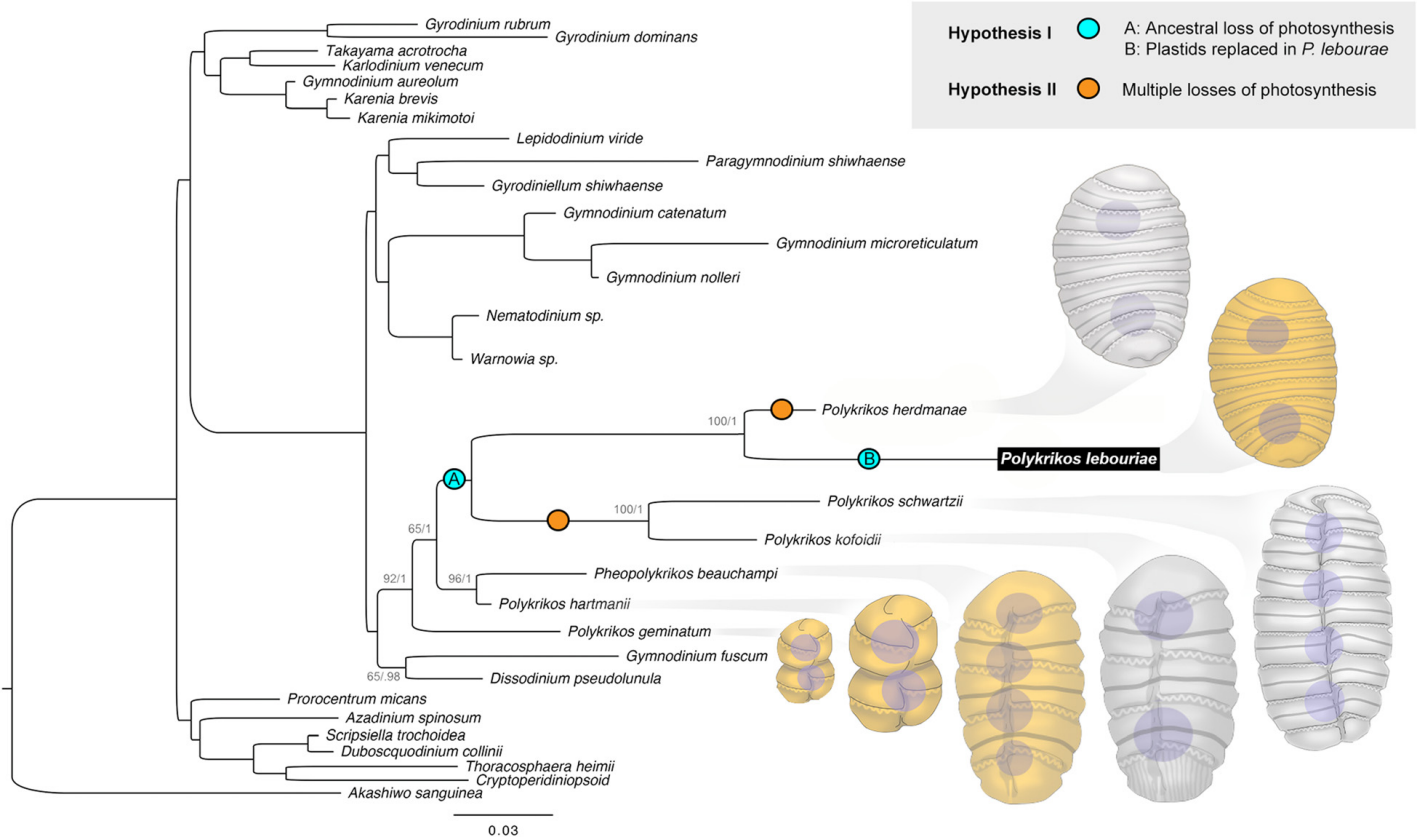
Maximum likelihood (ML) tree inferred from the 31-taxon alignment (1,915 unambiguously aligned sites) of concatenated small and large ribosomal rDNA sequences using the GTR + Γ substitution model. Bootstrap support values 65 or higher and Bayesian posterior probabilities are listed above each branch The illustrations depict the pseudocolonies of polykrikoid species; orange indicates a photosynthetic pseudocolony, and gray indicates a non-photosynthetic pseudocolony

Polykrikoid dinoflagellates are a distinctive group of uncultivated eukaryotes, including heterotrophic, photosynthetic, and mixotrophic species. They are recognized by their large, multinucleated cells or “pseudocolonies,” and include species that regulate harmful algal blooms by grazing on toxic dinoflagellates [7, 8]. Early-branching polykrikoids, such as *Polykrikos geminatum* and *P. hartmanii*, have plastids with three membranes and triple-stacked thylakoids that are characteristic of the secondary peridinin-type plastids of most dinoflagellates [9, 10] (Fig. 1). The plastids of *P. lebouriae*, however, are unusual; they are reported to be enveloped by only two membranes, a trait that is more consistent with primary plastids, and to contain double-stacked thylakoids similar to those found in haptophytes and diatoms [11]. *P. lebouriae* also has a conspicuous phylogenetic position, as a plastid-bearing mixotroph nested among three heterotrophic species (*P. herdmanae*, *P. schwartzii* and *P. kofoidii*) (Fig. 1). Leander and Hoppenrath (2007b) interpreted this as evidence of either multiple losses of photosynthesis among *P. herdmanae*, *P. schwartzii* and *P. kofoidii*, or a single loss at the base of this group, followed by acquisition of tertiary plastids in *P. lebouriae* from a diatom or haptophyte [12]. These hypotheses remain untested, as several attempts to cultivate *P. lebouriae* have been unsuccessful (Aika Yamaguchi, Mona Hoppenrath, personal communication), and PCR amplification of plastid genes in *P. lebouriae* has consistently failed with PCR primers used successfully in other taxa.

Multiple displacement amplification is a powerful tool for whole-genome amplification from small amounts of template DNA, but it is nonspecific and therefore prone to contamination [13]. We employed this technique to amplify a partial plastid genome of *Polykrikos lebouriae*, and supplemented this with a dinoflagellate-specific transcriptomic approach, both to ensure that our plastid amplification did not stem from non-dinoflagellate environmental contaminants (i.e.; diatoms, haptophytes or other algae that share the same habitat as *P. lebouriae*), and to test whether the plastids are functionally integrated into the cell (ie; if the nucleus expresses plastid-targeted genes) rather than being simply retained as kleptoplastids. We synthesized cDNA from single *P. lebouriae* cells, which we primed for PCR with a 21 bp spliced leader sequence specific to dinoflagellates, via SLPCR. Previous researchers have established the effectiveness of SLPCR for amplifying dinoflagellate transcripts from a large volume of wild-caught plankton [14] or coral tissue [15], and this is the first study to apply SLPCR at the scale of single cells (Additional file 1).

MDA and SLPCR allowed us to illuminate regions of the plastid genome in *P. lebouriae* as well as nuclear gene expression. In concert, these methods provided evidence of the presence and provenance of functional plastids *in P. lebouriae*, and allowed us to test hypotheses for plastid evolution in this uncultivated group.

## Results

### Genes for plastid-targeted proteins obtained from a single-cell transcriptome

*Polykrikos lebouriae* was identified by morphology in marine sand, and single cells were manually isolated for transcriptome and genome sequencing (see below). The identification was confirmed by comparing DNA fragments of HSP90 and LSU rRNA genes from single cell sequence data to sequences obtained from previous isolates of *Polykrikos lebouriae* (LSU rDNA sequences shared 96.8 % identity and HSP90 sequences shared 98.9 % identity). In order to sequence the transcriptome, transcripts were reverse transcribed and amplified using dinoflagellate spliced leader and polyA primers. For transcripts over 500 base pairs, the average length, after assembly, was 725 base pairs (Table 1). Estimates of genome coverage were not possible as no sequenced genome is available for *Polykrikos lebouriae* or any species within its more inclusive clade (i.e., the Gymnodiniales).

**Table 1:** Nuclear-encoded plastid-targeted genes transcripts (>600 bp) expressed by *Polykrikos lebourae*. Identifies were assigned using BLASTX queries against all proteins in Genbank

Genbank Predicted Plastid-Targeted Proteins	#	Top Hit	E value	Coverage	Identity
chloroplast ferredoxin	8	*Alexandrium fundyense*	6.00E-41	65 %	**68 %**
chloroplast light harvesting complex protein	8	*Symbiodinium sp.*	1.00E-60	45 %	**61 %**
chloroplast acyl carrier protein	5	*Heterocapsa triquetra*	2.00E-31	54 %	**65 %**
plastid C1 class II fructose bisphosphate aldolase	4	*H. triquetra*	0	68 %	**85 %**
chloroplast carbonic anhydrase	4	*H. triquetra*	7.00E-72	53 %	**71 %**
chloroplast ATP synthase subunit C	4	*A. affine*	2.00E-32	60 %	**100 %**
chloroplast phosphoribulokinase	3	*Lingulodinium polyedrum*	7.00E-164	93 %	**79 %**
chloroplast ribose-5-phosphate isomerase	3	*H. triquetra*	5.00E-96	71 %	**69 %**
chloroplast peridinin-chlorophyll a-binding protein precursor	2	*A. tamarense*	5.00 E-103	99 %	**82 %**
chloroplast ATP synthase gamma subunit	2	*H. triquetra*	3.00E-125	87 %	**57 %**
chloroplast ferredoxin-NADP{+) reductase	1	*H. triquetra*	1.00E-167	73 %	**70 %**
chloroplast photosystem I, subunit III	1	*H. triquetra*	2.00E-42	43 %	**51 %**
chloroplast photosystem II 12 kDa extrinsic protein	1	*H. triquetra*	7.00E-42	69 %	**62 %**
chloroplast photosystem I subunit XI	1	*H. triquetra*	1.00E-85	74 %	**50 %**

**Fig. 2:**
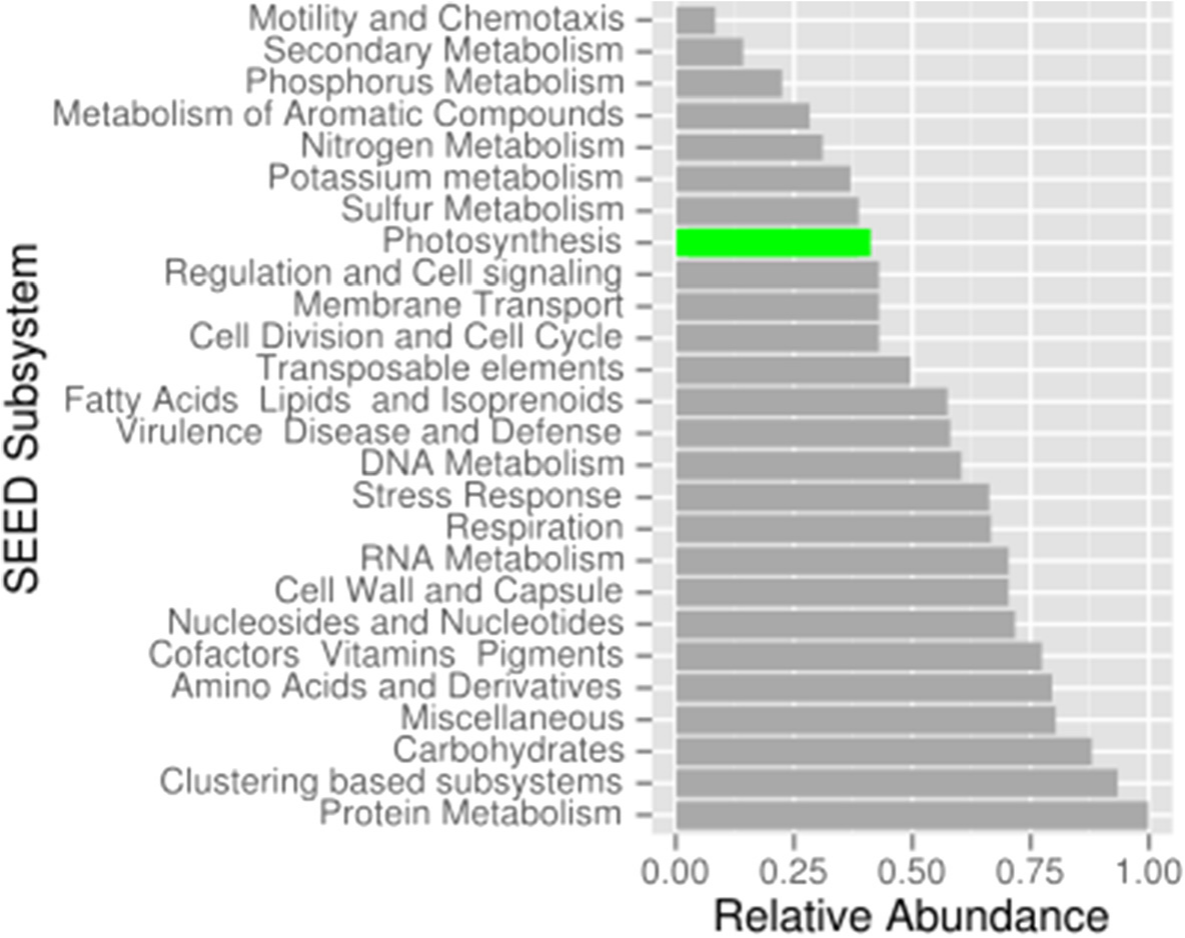
Transcripts expressed by a single cell isolate of *Polykrikos lebouriae*. Transcripts are ranked from values 0 to 1 in abundance, and annotated according to Level 1 Subsystem hierarchical classification in MG-RAST. Predicted photosynthetic transcripts are shown in green

SLPCR amplified a diverse array of nuclear transcripts from *P. lebouriae* (Fig. 2), suggesting that the cell expressed genes spanning a broad range of functions, including photosynthesis. Fourteen transcripts over 600 base pairs long were associated with photosynthesis, and all were most closely related to dinoflagellates (Table 1). These were all nucleus-encoded, plastid-targeted genes, supporting the presence of a plastid that is functionally integrated with the cell. Among these transcripts were two peridinin-chlorophyll a-binding precursor proteins, which are restricted to the peridinin-type plastids of dinoflagellates.

### Plastid-encoded genes obtained from single cell genomic data

**Fig. 3:**
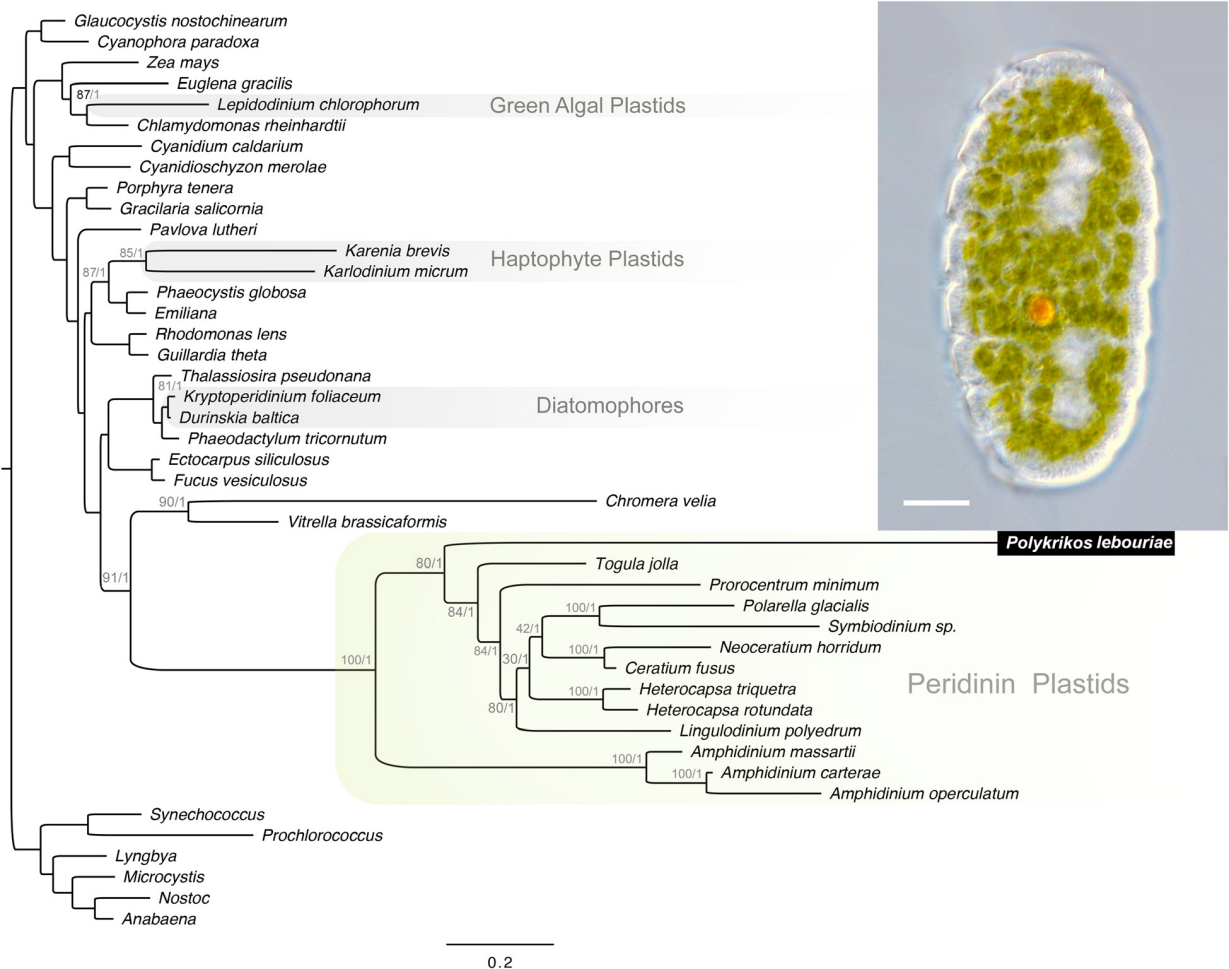
Maximum likelihood (ML) tree inferred from the 44-taxon alignment (1,595 unambiguously aligned amino acids) of concatenated plastid genes PsaA, PsbC, and AtpA using the PROTGAMMA model in RaxML. Bootstrap support values 65 or higher and Bayesian posterior probabilities are listed above each branch. The inset depicts a differential image contrast (DIC) micrograph of the pseudocolony of *Polykrikos lebouriae* used for single-cell transcriptomics; this cell was undergoing mitosis when the image was captured (scale bar = 10 μm)

In order to examine the genome of the plastid itself, we also sequenced a genomic library created by multiple displacement amplification (MDA) from a single cell. Unlike the dinoflagellate specificity achieved through SLPCR, our total genomic amplification through MDA yielded a majority of reads (64 %) from bacteria, with most of the remainder (34 %) stemming from dinoflagellates, and a small fraction of viral or uncertain provenance (2 %). Of the eukaryotic reads, 5 % were from plastids, with most other reads originating from the massive dinoflagellate nuclear genome. Bacterial sequences were primarily from delta proteobacteria, specifically *Francisella* sp., which is known from cosmopolitan marine and freshwater strains as well as symbiotic strains found among animals and protists [16, 17]. The eukaryotic sequences were most similar to dinoflagellates, as expected, and we identified and assembled three protein-coding genes from the plastid photosystem that are universally plastid-encoded: complete PsaA and PsbC genes and a partial plastid AtpA gene. After confirming the identity of each plastid photosystem gene using molecular phylogenetic analyses of the individual proteins (Additional file 3-5, Figures 3–5), the three proteins were concatenated and added to a 44-taxon alignment containing diverse dinoflagellates and other photosynthetic eukaryotes. Both Bayesian analysis and maximum likelihood methods demonstrated that the *P. lebouriae* plastid sequences branch with homologues from peridinin-type plastids of other unarmored dinoflagellates (Fig. 3). The sequences from *P. lebouriae* were highly divergent, but branched with strong support after the *Amphidinium* clade and before the clade consisting of *Togula jolla* and all armored dinoflagellates. Thus, the phylogenetic relationships inferred from the alignment of concatenated plastid-protein sequences are generally consistent with the placement of *P. lebouriae* as inferred from ribosomal gene sequences (Figs. 1, 3).

## Discussion

The majority of microbial species are not available in culture, and therefore the application of single cell methods at the genomic level is highly desirable [18]. In this case, we used both single cell transcriptomics and single cell genomics to investigate the biology of plastids in *P. lebouriae* and test hypotheses for their origin, which were otherwise difficult to resolve. Single-cell spliced-leader transcriptomics was particularly powerful, and using this method we were able to obtain a diversity of nuclear-encoded transcripts from *P. lebouriae*, despite the presence of environmental contamination from bacteria (as seen in the MDA results) and potentially even other non-dinoflagellate eukaryotes. Both nucleus-encoded transcripts and plastid-encoded genes consistently demonstrated that *P. lebouriae* is photosynthetic, with all photosynthesis related genes and transcripts grouping with those found in dinoflagellates with peridinin-type plastids, including a protein with the principle function of binding the pigment peridinin. No abnormalities were seen in the plastid targeting sequences to suggest that *P. lebouriae*, which we found to bear typical Type I and II presequences (Additional file 6: Figure S6), as described in dinoflagellates with triple membrane bound peridinin plastids [20–22]. Thus is it unclear whether the two plastid membranes reported by Hoppenrath and Leander were an accurate interpretation, a misinterpretation, or an artefact.

### Peridinin plastids in *Polykrikos lebouriae*

While we cannot falsify the possibility of transient or hidden plastids in some polykrikoids, our findings are contrary to the hypothesis that *Polykrikos lebouriae* acquired photosynthesis from diatoms or haptophytes and support the presence of peridinin-type plastids in *P. lebouriae*. The most parsimonious source for these plastids is direct inheritance from ancestral polykrikoids. Polykrikoid phylogeny, though lacking strong support at some deeper nodes, shows an unequivocal sisterhood between *P. lebouriae* and heterotrophic *P. herdmaniae—*which necessitates a recent loss of photosynthesis in *P. herdmaniae*. A second loss is evident in the *P. kofoidii - P. schwartzii* clade, as they are strongly supported sister lineages, and therefore represent a loss independent from that found in *P. herdmaniae*.

### Hypothesis for polykrikoid plastid evolution

Several losses of photosynthesis have previously been established in dinoflagellates [19], primarily among parasitic stem groups or within groups of questionable monophyly (e.g., the Gymnodiniales). Interestingly, multiple losses of photosynthesis appear to have occurred within polykrikoids alone, and the evolutionary reasons for this are worth considering. A prominent trend in polykrikoid evolution is a gradual increase in size [12] (Fig. 1). This makes polykrikoids effective predators, as they are able to consume groups of dinoflagellates linked in defensive chain formations [23]. Yet size is known to make photosynthesis less effective for single cells, as chloroplast self-shading increases, and absorptive surface area diminishes relative to volume [24]. As a mixotroph, *P. lebouriae* is known to prey on other dinoflagellates (Aika Yamaguchi, personal communication), and our isolate possessed extrusive organelles such as nematocysts and taeniocysts. The presence of such specialized predatory features, as well as mixotrophy and large cell size, may have predisposed polykrikoids to multiple losses of photosynthesis, as seen in *P. herdmaniae*, a sister species that shares the same habitat as *P. lebouriae* [11]. Factors allowing the loss of photosynthesis probably vary by lineage, as losses have also occurred among smaller free-living and parasitic dinoflagellates.

Dinoflagellates are fascinating models for the study of organelle evolution, for in addition to plastid loss, they represent many stages in the process of plastid acquisition [1]. For instance, “dinotoms” house virtually intact diatom symbionts [4]; *Pfiesteria piscicida* scavenges temporary kleptoplastids with a limited lifespan [25]; and *Karenia* and *Karlodinium* have haptophyte-derived plastids that are nearly as integrated as native peridinin-type plastids [26, 27]. The challenge in studying endosymbiosis at the earlier stages (where the symbiont retains some genetic autonomy) lies partly in differentiating symbiont-expressed transcripts from those of the host. SLPCR circumvents this problem by ensuring dinoflagellate specificity [14, 15]. In the future, this approach is expected to grant insight into endosymbioses in other uncultivated dinoflagellates, such as the plastid or symbiont-bearing Noctilucales [28] and new lineages of dinoflagellates with cryptophyte or pelagophyte symbionts [29–31].

### Conclusions

Understanding trends in the evolution of microbial eukaryotes will require a synthesis of ecology, phylogenetics, and genomics—the last of which has been particularly limited in its applications to uncultivated groups. While SLPCR has previously been applied to bulk RNA samples [14, 15, 32], we show here that it is applicable to single cells. In principle, this method is applicable to any system with uniform spliced leaders, as found in dinoflagellates, euglenids, kinetoplastids, and a growing number of invertebrates [33]. SLPCR shows promise not only in avoiding contaminants in environmental isolates, but in capturing gene expression of a single cell at a given point in time, such as stages of the cell cycle, cells perturbed my experimental stimuli, or simply cells in the dynamism of their natural habitats.

## Methods

### Collection of organisms

Samples of the upper 1-cm of marine sand were collected during low tide from the mid intertidal zone in Cannon Beach, Oregon during early October. Within 36 h, the samples were transported to the University of British Columbia, kept in an open dish of moist sand and exposed to natural day/night rhythms. Uhlig's seawater ice method was used to draw cells from the sand into a petri dish, where they were collected individually by micropipette [34]. Cells were visually identified based on the presence of two-nuclei, eight zooid segments, and plastids, and later confirmed through analysis of the LSU ribosomal gene. To reduce the chance of genetic contamination from prey, we selected cells of *P. lebouriae* in which no food vacuoles were evident. Pseudocolonies of *P. lebouriae* were washed five times, once in filtered seawater, twice in PBS buffer, and twice in distilled nuclease-free water. For transcriptomics, a single cell (Fig. 3, inset) was processed for RNA extraction immediately; other single cell isolates were frozen at −80 °C and thawed later that week for genomics processing using MDA.

### Single cell transcriptomics

For cell lysis, 0.5 μl of proteinase K was added to the tubes, followed by incubation at 65 °C for 10 min, and denaturation at 90 °C for 2 min, then rapid cooling at 4 °C. First strand cDNA was primed using a GeneRacer OligodT primer (1 μl at 10 μM) RNAse Out (0.5 μl), dNTPs – (2 μl at 10 μM) and incubated at 65 °C for 5 min. After this annealing step, DTT (1 μl at 10 μM), Rnase Out (0.5 μl), and 1 μl of Superscript III reverse transcriptase (Life Technologies, Carlsbad CA) were added and incubated according to the manufacturer’s protocol, allowing for reverse transcription, along with T4 gene 32 (0.5 μl) to maximize contact between the reverse transcriptase and RNA template. Afterwards, DNA/RNA hybrids were removed with 1 μl of RNAseH, incubated 37 °C for 20 min.

Polyadenylated transcripts were amplified with a a GeneRacer 3’ nested primer (5’-CGCTACGTAACGGCATGACAGTG-3’), and dinoflagellate specificity assured with the a dinoflagellate spliced-leader primer (5’-TCCGTAGCCATTTTGGCTCAAG-3’) and (Life Technologies, Carlsbad CA). Thermocycling proceeded through a “touchdown PCR” program, as this was effective for Zhang *et al.* (2007). This program progressed through 95 °C for 20 s, 72 °C for 2.5 min for 5 cycles; 95 °C for 20 s, 65 °C for 30 s, 72 °C for 2 min for 5 cycles; 95 °C for 20 s, 60 °C for 30 s, 72 °C for 2 min for 5 cycles; and 95 °C for 20 s, 58 °C for 30 s, 72 °C for 2 min for 25 cycles. Because we were amplifying from a single cell, our PCR reaction program had ten more amplification steps than that of Zhang et al. (2007). In order to reduce amplification bias, we divided each SLPCR reaction into eight sub-reactions, which ran in parallel, and were pooled at the end. Reads were quality checked using a Bioanalyzer (Agilent, Santa Clara, CA).

### Single cell genomics

A frozen cell was thawed on ice then lysed as above. Multiple Displacement Amplification (MDA) was carried out using the Repli-G mini kit (Qiagen, Venlo, Linburg, Netherlands) as per manufacturer’s instructions, at the maximum recommended time of 15 hours. The reaction was divided into four sub-reactions to minimize amplification bias and then pooled at the end. Genomic DNA was amplified non-specifically, including the plastid genes of *P. lebouriae*.

### Sequencing, assembly and annotation

DNA for libraries was sheared to ~300-400 bp by a Covaris Ultrasonicater (M220) using the manufacturer’s protocol (Covaris, Woburn, MA). Libraries were indexed with TruSeq^Tm^ adapter barcodes using Lucigen NxSeq library prep without PCR enrichment to avoid amplification bias. Libraries were washed with two rounds of AMPureXP magnetic beads (Beckman Coulter, Danvers, MA) at a beads to DNA ratio of 0.8:1 to remove free adapters by size screening. To ensure sufficient adapter ligation, a sample of the libraries were tested with real-time qPCR (primed to the library indexes), and measured against a digital standard curve [35]. Libraries were screened for purity using a Nanodrop (ThermoFisher, Wilmington, DE) and length and purity using a Bioanalyzer HighSens DNA chip (Agilent, Palo Alto, CA). Libraries were sequenced with 250 bp paired-end reads on an Illumina MiSeq (GenoSeq UCLA Los Angeles, CA).

A phiX library was used as a quality standard during sequencing. From the output sequences, phiX was screened and removed, paired ends were merged (if overlapping >30 bp), and non-overlapping reads were interleaved. Merged reads were checked for a minimum Qscore (Q > 30). De novo assembly was performed with Ray [36] using a variety of kmer sizes, with 31 chosen as the optimal kmer size for assembling our genomic reads and 53 for reads from SLPCR. Resulting contigs were uploaded to the MG-RAST server, which performed automated annotations and protein predictions [37].

### Multiple sequence alignments

Several alignments were constructed in this study for molecular phylogenetic analysis. For analysis of ribosomal genes, we concatenated small and large subunit rDNA sequences, and aligned them across 31 unarmoured dinoflagellates, with *Akashiwo sanguinea* as the outgroup. This nucleotide alignment consisted of 1,915 unambiguously aligned sites, once gaps and ambiguously aligned regions were removed. A second alignment was assembled for LSU rDNA sequences alone, in order to confirm that the single cell isolate was *Polykrikos lebouriae*. This alignment included 25 dinoflagellate taxa, with 1,229 unambiguously aligned bases (Additional file 2).

The remaining alignments were for protein analyses, translated plastid genes PsaA, PsbC and AtpA. Predicted proteins were aligned with MUSCLE, followed by removal of gaps and ambiguously aligned bases. Using 100 boostraps of RAxML and the substitution model PROTGAMMA, preliminary trees were generated from MUSCLE [38] alignments of 715 amino acids for PsaA (Additional file 3: Figure S3), 453 aligned amino acids for PsbC (Additional file 4: Figure 4), and 427 aligned amino acids for AtpA (Additonal file 5 Figure 5). Having validated these proteins as dinoflagellate plastid-type proteins, we manually concatenated these three alignments into a supermatrix with 1,595 unambiguously amino acids for the final analysis. This alignment incorporated 44 taxa, including representatives of all major groups of photosynthetic eukaryotes, including glaucophytes, red algae, green algae, land plants, haptophytes cryptophytes, stramenopiles, dinoflagellates with peridinin-type plastids, as well *Lepidodinium*, *Karenia*, *Karlodinium*, *Durinskia*, *Kryptoperidinium* and six species of cyanobacteria. We chose dinoflagellate taxa for which two or more of the plastid proteins were available in Genbank or CAMERA. Among dinoflagellates, *Symbiodinium*, *Togula*, *Lingulodinium*, *Lepidinodinium*, *Kryptoperidinium*, *Durinskia*, *Aphidinium carterae*, *Heterocapsa rotundata*, and *Polykrikos lebouriae* had all three proteins, and dinoflagellates with two proteins were incorporated as they led to higher resolution of the dinoflagellate relationships.

### Molecular phylogenetic analyses

Maximum likelihood analysis was run with 1,000 bootstraps using RAxML and PROTGAMMAJTT or GRTGAMMA substitution models for protein and nucleotide sets, respectively [39]. Bayesian posterior probabilities were calculated for all alignments using the following parameters on the program MrBayes 3.2.2 (GTR [Lset nst = 6]; gamma distribution [of rate among sites]; and Monte Carlo Markov Chains [starting trees = 4; heating (nchains = 4), default temperature = 0.2; generations = 6,000,000; sample frequency = 100; prior burn-in = 500,000 trees] [40, 41].

### Presequence analysis of plastid targeted genes

The n-terminal region of nuclear encoded, putatively plastid targeted genes was analysed for signal peptides using the Hidden Markov Model of SignalP3.0 [42, 43] using default settings. Transmembrane helices were predicted using TMHMM v.2.0 [44], and their hydrophobicity scores were calculated with the Kyte-Doolittle amino acid scale from Protscale (http://web.expasy.org/protscale/, last accessed April 22, 2015) using default settings. Protein sequences were manually aligned, in Mega 5.2.2 [45], and imported into Jalview [45], where the charge and hydrophobicity of amino acids were color coded.

### Data availability

All plastid genes that we sequenced and employed in our phylogenetic analysis were submitted to Genbank, with accession numbers KP259913 to KP259915. Nuclear-encoded plastid targeted proteins were given the accession numbers KR134302 to KR134310.

## Additional files

Additional file 1: Figure 1. Diagram of the basic steps in splice leader primed PCR (SLPCR). Additional file 2: Figure 2. Maximum likelihood (ML) tree inferred from a 25-taxon alignment of LSU rDNA sequences (1,229 unambiguously aligned bases) using the GTRGAMMA model in RAxML. Bootstrap support values and Bayesian posterior probabilities are listed above each branch. Additional file 3: Figure 3. Maximum likelihood (ML) tree inferred from a 39-taxon alignment of PsaA (photosystem 1 P700 chlorophyll a apoprotein A1) sequences (715 unambiguously aligned amino acids) using the PROTGAMMA model in RAxML. Bootstrap support values are listed above each branch. Additional file 4: Figure 4. Maximum likelihood (ML) tree inferred from a 44-taxon alignment of PsbC (photosystem II CP43 protein) sequences (453 unambiguously aligned amino acids) using the PROTGAMMA model in RAxML. Bootstrap support values are listed above each branch. Additional file 5: Figure 5. Maximum likelihood (ML) tree inferred from a 34-taxon alignment of AtpA (Atp synthase CF1 alpha chain) sequences (427 unambiguously aligned amino acids) using the PROTGAMMA model in RAxML. Bootstrap support values are listed above each branch. Additional file 6: Figure 6. Transcripts in *Polykrikos lebouriae* have plastid-targeted sequences typical of dinoflagellates with triple-membrane bound peridinin plastids. **A.** Class I transit peptides, each containing a transmembrane domain, which have been manually aligned, as have their “FVAP” motifs. Boxed proteins are previously published [22], typical Class I transit peptides from *Heterocapsa triquetra* (AAW79309, AY826901, AY826898), for comparison. All other proteins are from *P. lebouriae* (KR134302 – KR134310). The average hydrophobicity score of each column in the transmembrane domain and neighboring regions have been plotted above the alignment. Amino acid color code: Yellow = hydrophobic, blue = polar, green = negatively charged, red = positively charged. **B.** Class II transit peptides: These presequences lack a transmembrane domain, but contain the typical “FVAP” motif. For both classes of transit peptides, the “FVAP” cleavage-site (or nearly cleavage-site) motifs are listed to the right of the sequence alignment.

## Abbreviations

AtpA: Atp synthase CF1 alpha chain
LSU rDNA: Large subunit ribosomal DNA
MDA: Multiple displacement amplification
ML: Maximum likelihood
PsaA: Photosystem 1 P700 chlorophyll a apoprotein A1
PsbC: Photosystem II CP43 protein
SL: Spliced leader
SLPCR: Spliced leader polymerase chain reaction
SSU rDNA: Small subunit ribosomal DNA
