# Supplementary figures and images for: Single-cell transcriptomics using spliced leader PCR: Evidence for multiple losses of photosynthesis in polykrikoid dinoflagellates
Source: BMC Genomics. 2015 Jul 17;16(1):528. doi: 10.1186/s12864-015-1636-8 (PMC4504456; doi:10.1186/s12864-015-1636-8)

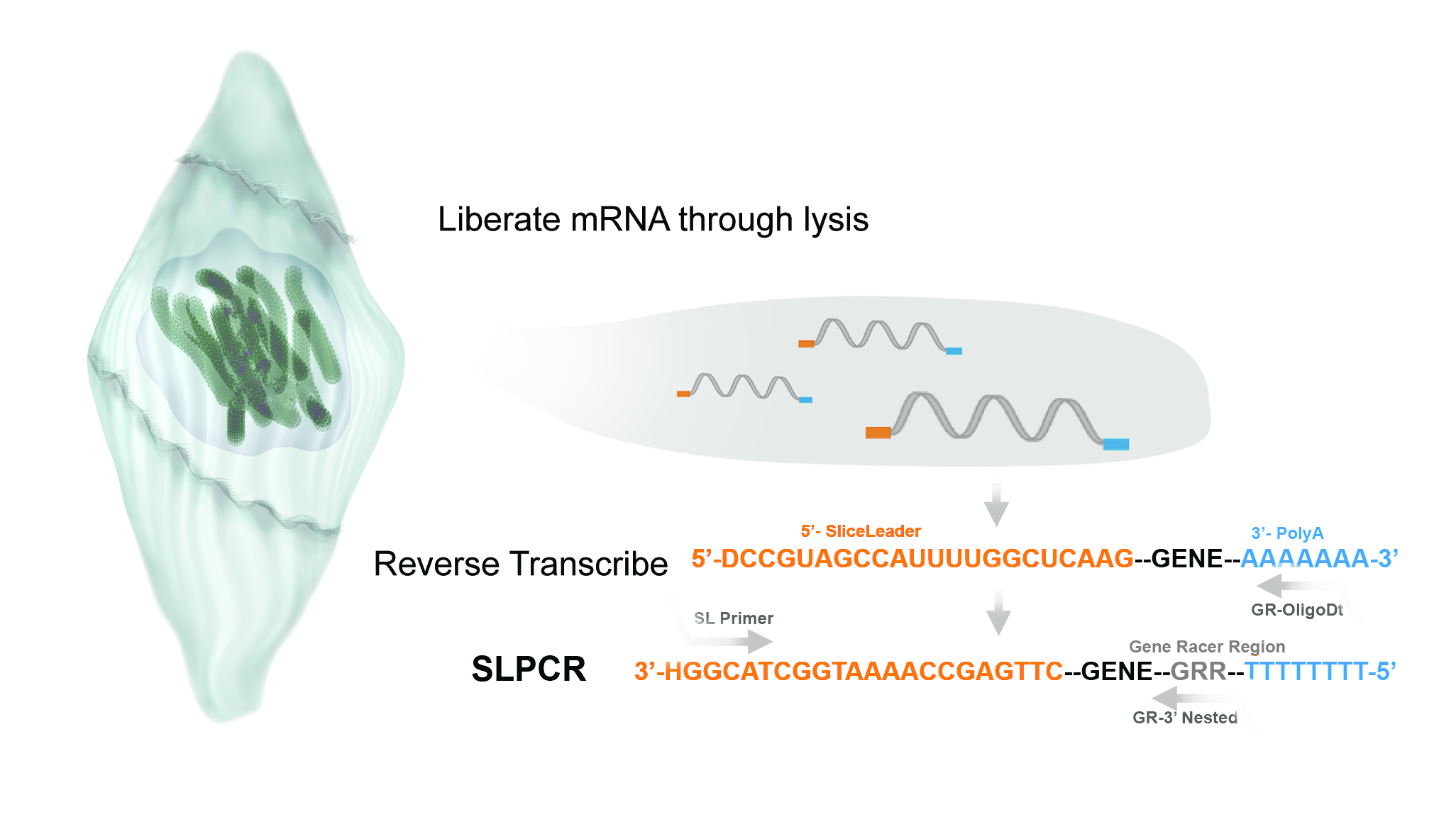

Supplement: Additional file 1: Figure 1. — Diagram of the basic steps in splice leader primed PCR (SLPCR). [file 12864_2015_1636_MOESM1_ESM.tiff]

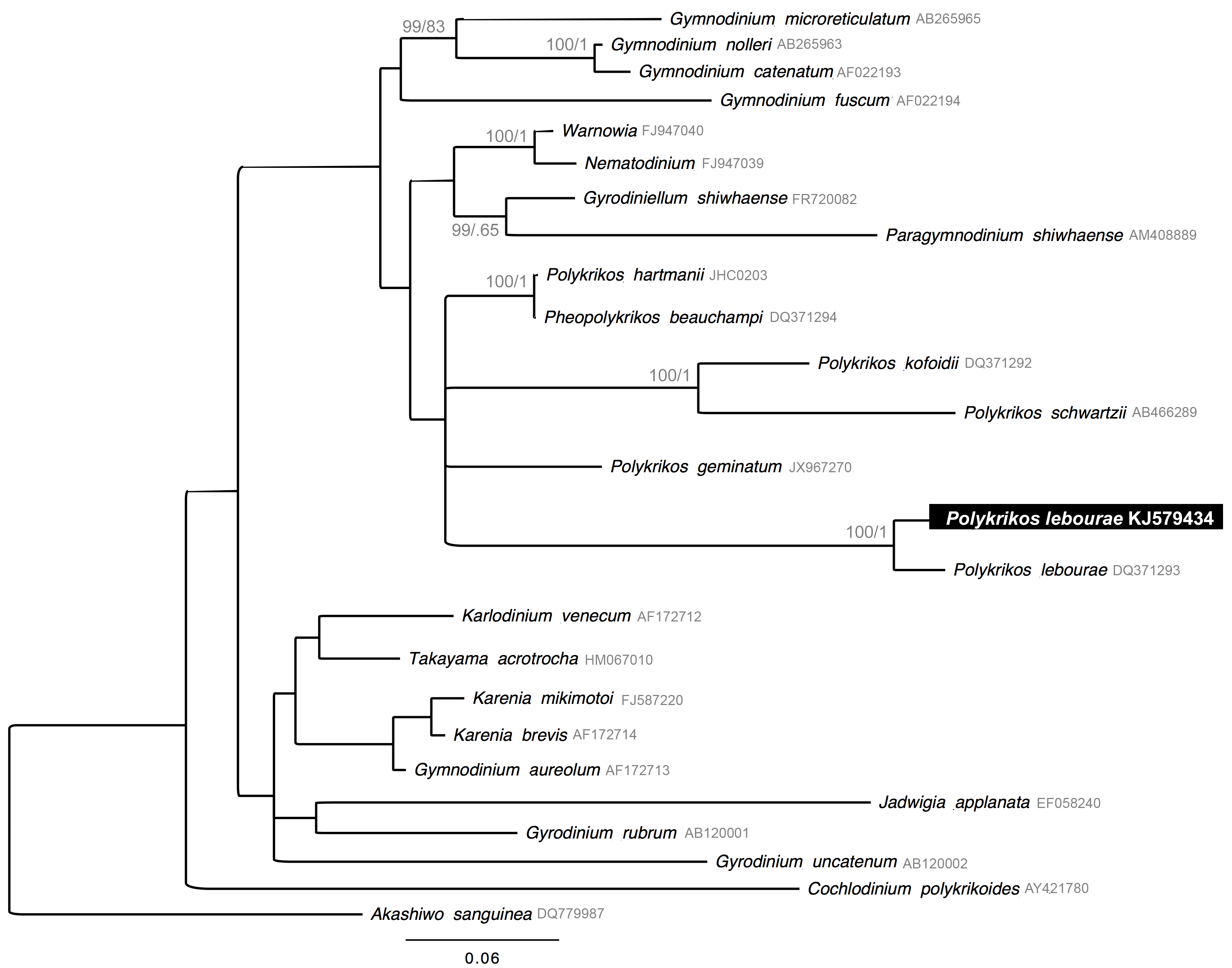

Supplement: Additional file 2: Figure 2. — Maximum likelihood (ML) tree inferred from a 25-taxon alignment of LSU rDNA sequences (1,229 unambiguously aligned bases) using the GTRGAMMA model in RAxML. Bootstrap support values and Bayesian posterior probabilities are listed above each branch. [file 12864_2015_1636_MOESM2_ESM.png]

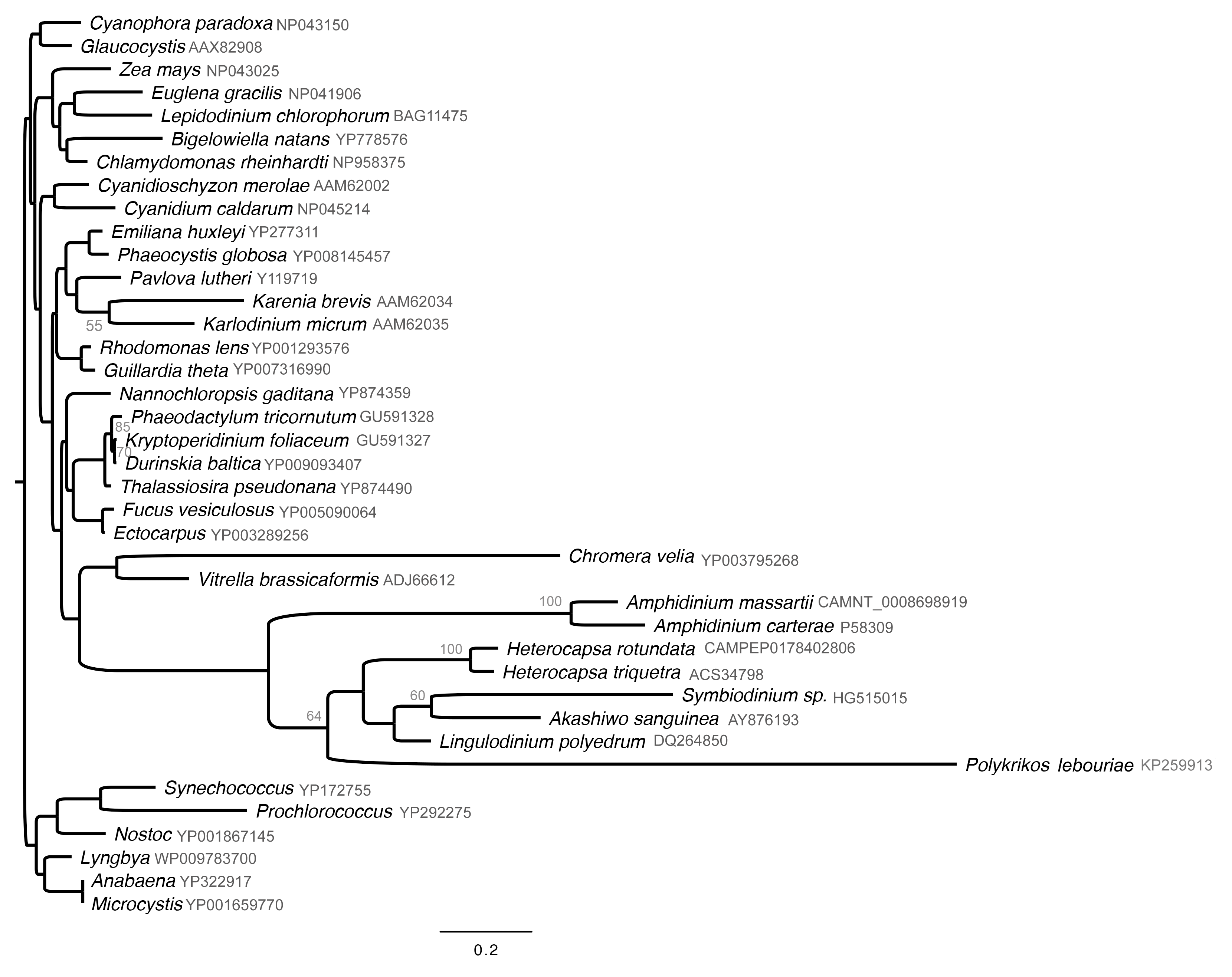

Supplement: Additional file 3: Figure 3. — Maximum likelihood (ML) tree inferred from a 39-taxon alignment of PsaA (photosystem 1 P700 chlorophyll a apoprotein A1) sequences (715 unambiguously aligned amino acids) using the PROTGAMMA model in RAxML. Bootstrap support values are listed above each branch. [file 12864_2015_1636_MOESM3_ESM.png]

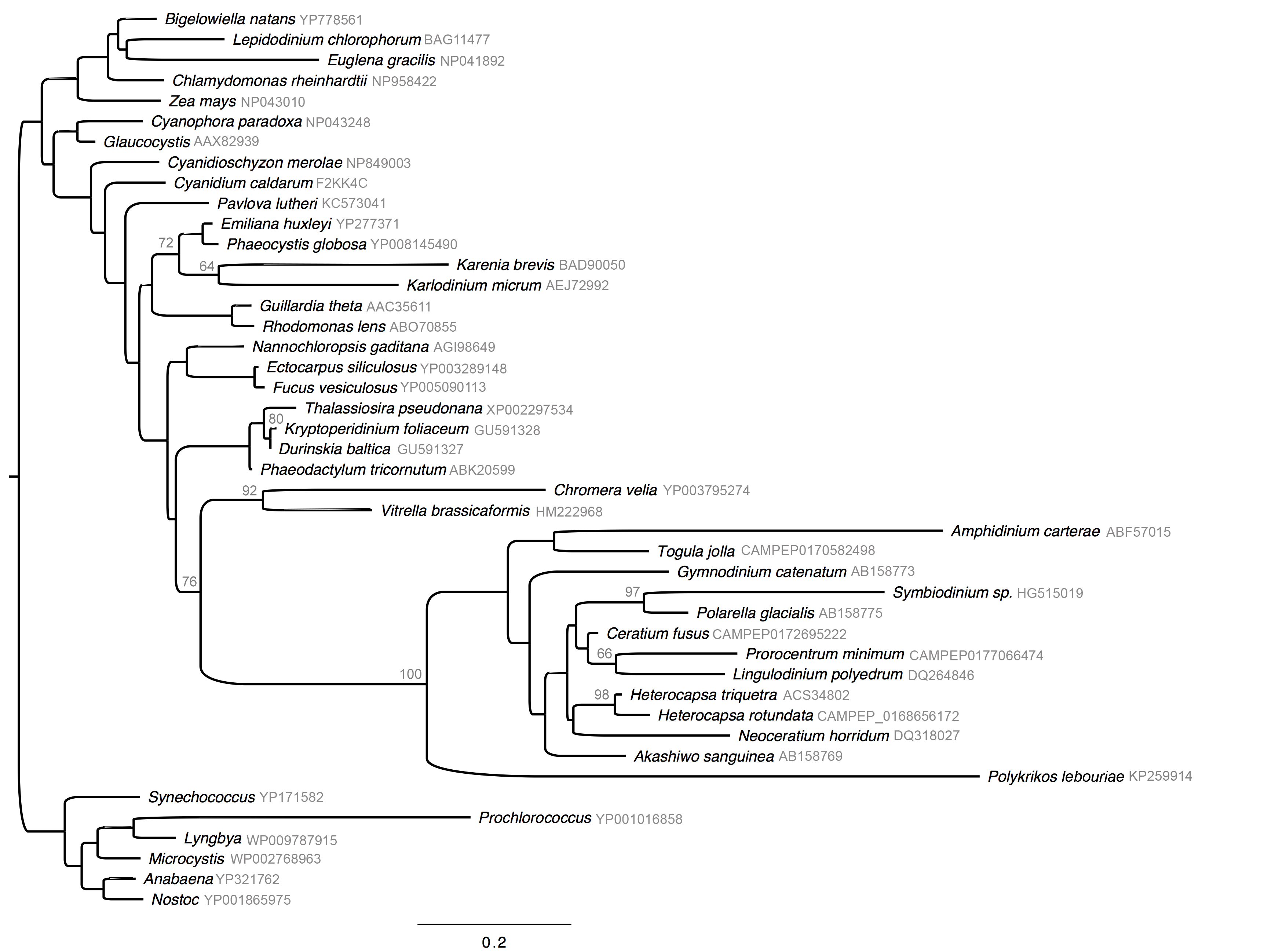

Supplement: Additional file 4: Figure 4. — Maximum likelihood (ML) tree inferred from a 44-taxon alignment of PsbC (photosystem II CP43 protein) sequences (453 unambiguously aligned amino acids) using the PROTGAMMA model in RAxML. Bootstrap support values are listed above each branch. [file 12864_2015_1636_MOESM4_ESM.png]

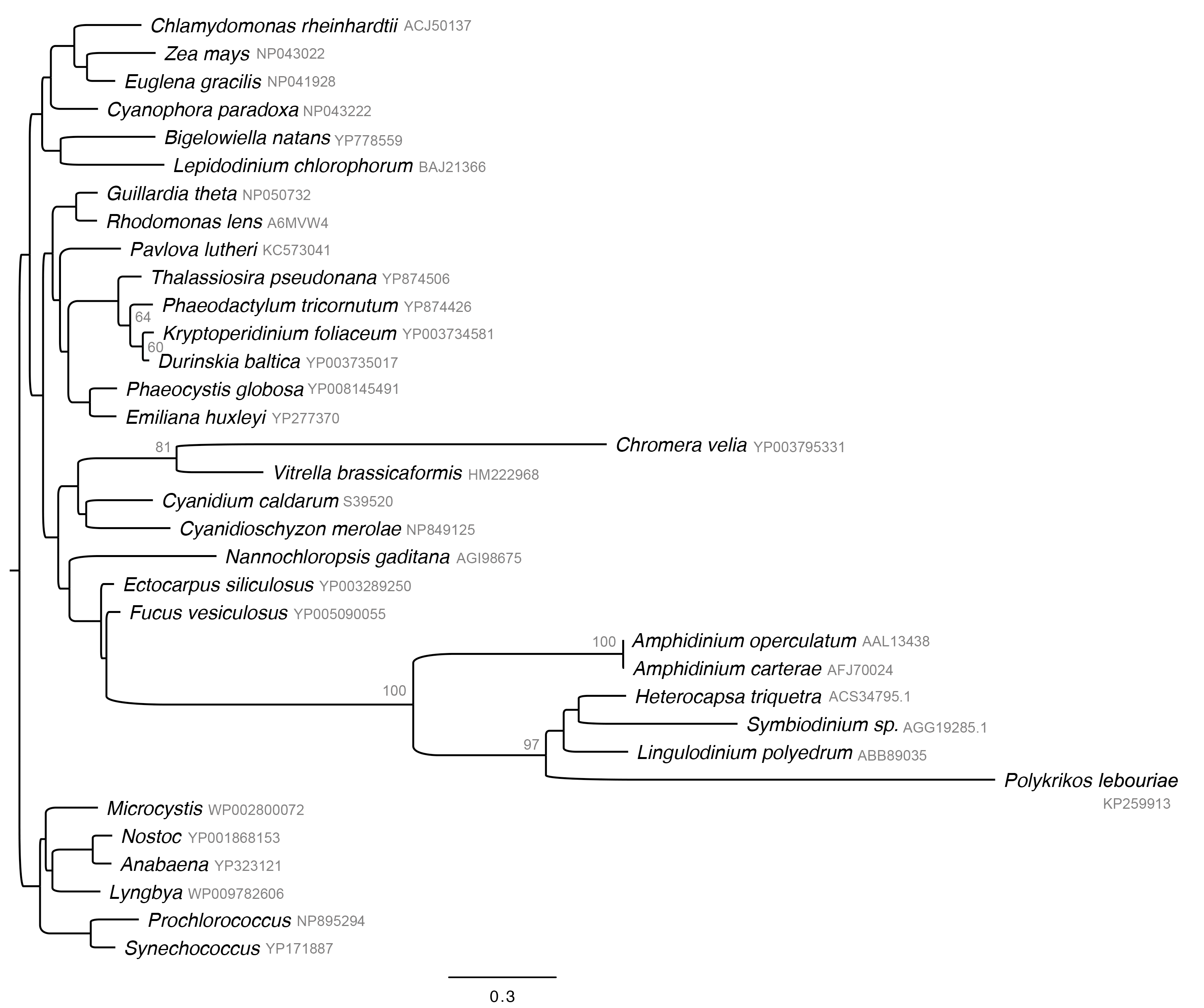

Supplement: Additional file 5: Figure 5. — Maximum likelihood (ML) tree inferred from a 34-taxon alignment of AtpA (Atp synthase CF1 alpha chain) sequences (427 unambiguously aligned amino acids) using the PROTGAMMA model in RAxML. Bootstrap support values are listed above each branch. [file 12864_2015_1636_MOESM5_ESM.png]

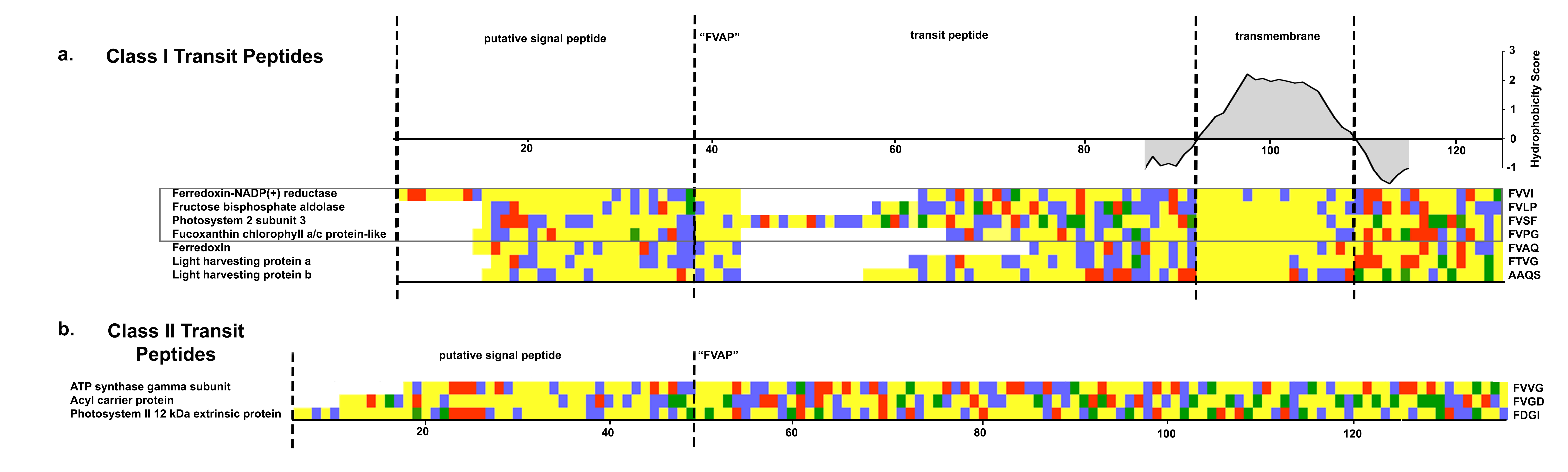

Supplement: Additional file 6: Figure 6. — Transcripts in Polykrikos lebouriae have plastid-targeted sequences typical of dinoflagellates with triple-membrane bound peridinin plastids. A. Class I transit peptides, each containing a transmembrane domain, which have been manually aligned, as have their “FVAP” motifs. Boxed proteins are previously published [22], typical Class I transit peptides from Heterocapsa triquetra (AAW79309, AY826901, AY826898), for comparison. All other proteins are from P. lebouriae (KR134302 – KR134310). The average hydrophobicity score of each column in the transmembrane domain and neighboring regions have been plotted above the alignment. Amino acid color code: Yellow = hydrophobic, blue = polar, green = negatively charged, red = positively charged. B. Class II transit peptides: These presequences lack a transmembrane domain, but contain the typical “FVAP” motif. For both classes of transit peptides, the “FVAP” cleavage-site (or nearly cleavage-site) motifs are listed to the right of the sequence alignment. [file 12864_2015_1636_MOESM6_ESM.png]
